# The effect of probiotics on gestational diabetes and its complications in pregnant mother and newborn: A systematic review and meta‐analysis during 2010–2020

**DOI:** 10.1002/jcla.24326

**Published:** 2022-03-03

**Authors:** Marzie Mahdizade Ari, Samane Teymouri, Tayebeh Fazlalian, Parisa Asadollahi, Roghayeh Afifirad, Mohammad Sabaghan, Fateme Valizadeh, Roya Ghanavati, Atieh Darbandi

**Affiliations:** ^1^ 440827 Department of Microbiology School of Medicine Iran University of Medical Sciences Tehran Iran; ^2^ 440827 Microbial Biotechnology Research Centre Iran University of Medical Sciences Tehran Iran; ^3^ 68106 Department of Microbial Biotechnology Tehran Science and Research Branch Islamic Azad University Tehran Iran; ^4^ Department of Microbiology Faculty of medicine Ilam University of Medical Sciences Ilam Iran; ^5^ 48439 Department of Microbiology School of Medicine Tehran University of Medical Sciences Tehran Iran; ^6^ Behbahan Faculty of Medical Sciences Behbahan Iran; ^7^ Department of Endodontics School of Dentistry Shahid Sadoughi University of Medical Sciences Yazd Iran

**Keywords:** gestational diabetes, pregnant women, probiotics, randomized controlled trial

## Abstract

This study was aimed to evaluate the effect of probiotics consumption on gestational diabetes (GD) and its complications in pregnant mother and newborn. The study was registered on PROSPERO (CRD42021243409) and all the enrolled articles were collected from four databases (Medline, Scopus, Embase, and Google Scholar) as randomized controlled trials (RCTs) from 2010 to 2020. A total of 4865 study participants from 28 selected studies were included in this review. The present meta‐analysis showed that the consumption of probiotics supplementation has the potential to decrease GD‐predisposing metabolic parameters such as blood glucose level, lipid profile, inflammation, and oxidative markers which may reduce GD occurrence among pregnant women.

## Graphical abstract

The effect of probiotic on gestational diabetes.

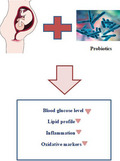

## 1 INTRODUCTION

Gestational diabetes (GD) refers to glucose intolerance in pregnant women at 24–28 weeks without a history of diabetes that result in hyperglycemia. Pregnant women who suffer from GD show various symptoms including unusual thirst, frequent urination, frequent infections, and weight gain.^1^ Lack of mobility and overweight are the main predisposing factors for GD which occurs in 17% of all pregnancies worldwide with a 10–100% increase in rate during the last 20 years. In recent years, Middle East, North Africa, and Europe have had the highest (12.9%) and lowest (5.8%) prevalence of GD, respectively.^2^, ^3^ GD can expose the health of the mother and the fetus at risk by complications including neonatal hypoglycemia, polycythemia, respiratory distress, hypocalcemia, gestational hypertension, pre‐eclampsia, increased cesarean section rate,^4^, ^5^ and type 2 diabetes mellitus (T2DM), as long‐term adverse outcome and the most commonly reported complication.^6^, ^7^ Therefore, it is important to prevent and control diabetes during pregnancy.

Defects in carbohydrate and lipid metabolism which some researchers have attributed to microbiome changes, as well as genetic disorders play an important role in the development of GD. During pregnancy, secretion of leptin and inflammatory cytokines such as IL‐6 and TNF‐α is directly related to oxidative damages and the levels of estrogen and progesterone as placental hormones, which in turn leads to increased insulin resistance and eventually the development of GD.^1^, ^5^, ^8^ Several approaches are suggested to control glucose levels during GD such as insulin injection, changes in lifestyle (diet and exercise), oral medications (e.g., metformin), and consumption of probiotics and vitamin D, although in many cases these strategies may not work.^6^, ^9^ Probiotics (*Lactobacillus* and *Bifidobacterium* spp.) are live microorganisms that, if prescribed properly, will have significant effects on human health.^10^ Probiotics have proven effective in many clinical applications such as the treatment of enterocolitis, diarrhea and cancers.^10^

Consumption of food products that carry probiotics, not only show to prevent food spoilage and growth of pathogens, but also have been effective in increasing the quality, taste, and appearance of foods. Probiotics included in the diet of broilers and laying animals led to an increase in growth of pigs, cows, broilers, and their products (egg yolks and milk production). In addition to increasing the percentage of proteins, probiotics in food industry leads to an improvement in color of meats and pH, reducing oxidative stress and lipid oxidation.^11^ Interestingly, dairy products are good source for containing probiotics (such ice cream containing *Lactobacillus acidophilus* and inulin) and improvement of gastrointestinal tract.^12^ Gut health was achieved by using probiotics in foods that results in an increase number of probiotic bacteria, reduces the number of fecal pathogens such as *coliforms* and *staphylococci* spp, and improves the quality of feces (in terms of the presence of water and reduced acidity).^13^

Some study suggests that probiotics are able to overcome insulin resistance in pregnant women with GD by consuming the blood sugar as energy source, improving lipid metabolism in the gut, increasing glutathione (GSH) levels, and reduction of the inflammatory biomarkers like high sensitive C‐reactive protein (hs‐CRP) and oxidative stress.^5^, ^7^ Through pregnancy, the inflammatory conditions of the body affect the number of bacteria such as *Bifidobacterium* and *Bacteroides* and their balance in the body, while probiotics are able to induce gut microbiome to reduce the effects of metabolic defects. In addition, microbial imbalance in women with GD which is known as “Gut microbiome dysbiosis,” includes an increase in the number of *Bacteroides* spp. and a decrease in the number of *Bifidobacterium* spp. and is the main cause of overweight among pregnant woman, while their hypertension which may be related to low dietary fiber.^9^, ^14^, ^15^

Moreover, the use of probiotics during pregnancy is not harmful and is well tolerated in the body.^4^ Although choosing the best probiotic and the optimal dose for the treatment of GD requires more studies, *Bifidobacterium* and *Lactobacillus* spp. have been commonly used in studies with more than 10^7^ CFU/ml daily, as the suggested dose needed to achieve desirable results on the reduction of metabolic dysfunction.^8^, ^16^ Here, the aim of this study was to investigate the inhibitory effects of probiotics supplementation on GD among pregnant women based on Randomized Controlled Trial (RCT) studies during in the last 10 years (2010–2020).

## 2 MATERIALS AND METHODS

### 2.1 Guidelines

This systematic review and meta‐analysis was performed according to the PRISMA 2020 guidelines (File S1).^17^ The study was registered on PROSPERO (CRD42021243409).

### 2.2 Information sources and search strategy

Data from the four international information databases Medline, Scopus, Embase, and Google Scholar were searched during 2010–2020. The search strategy was based on the combination of the following terms: “gestational diabetes” and “probiotics.” The search items in each database are also available in the File S2.

### 2.3 Inclusion and exclusion criteria

**FIGURE 1:**
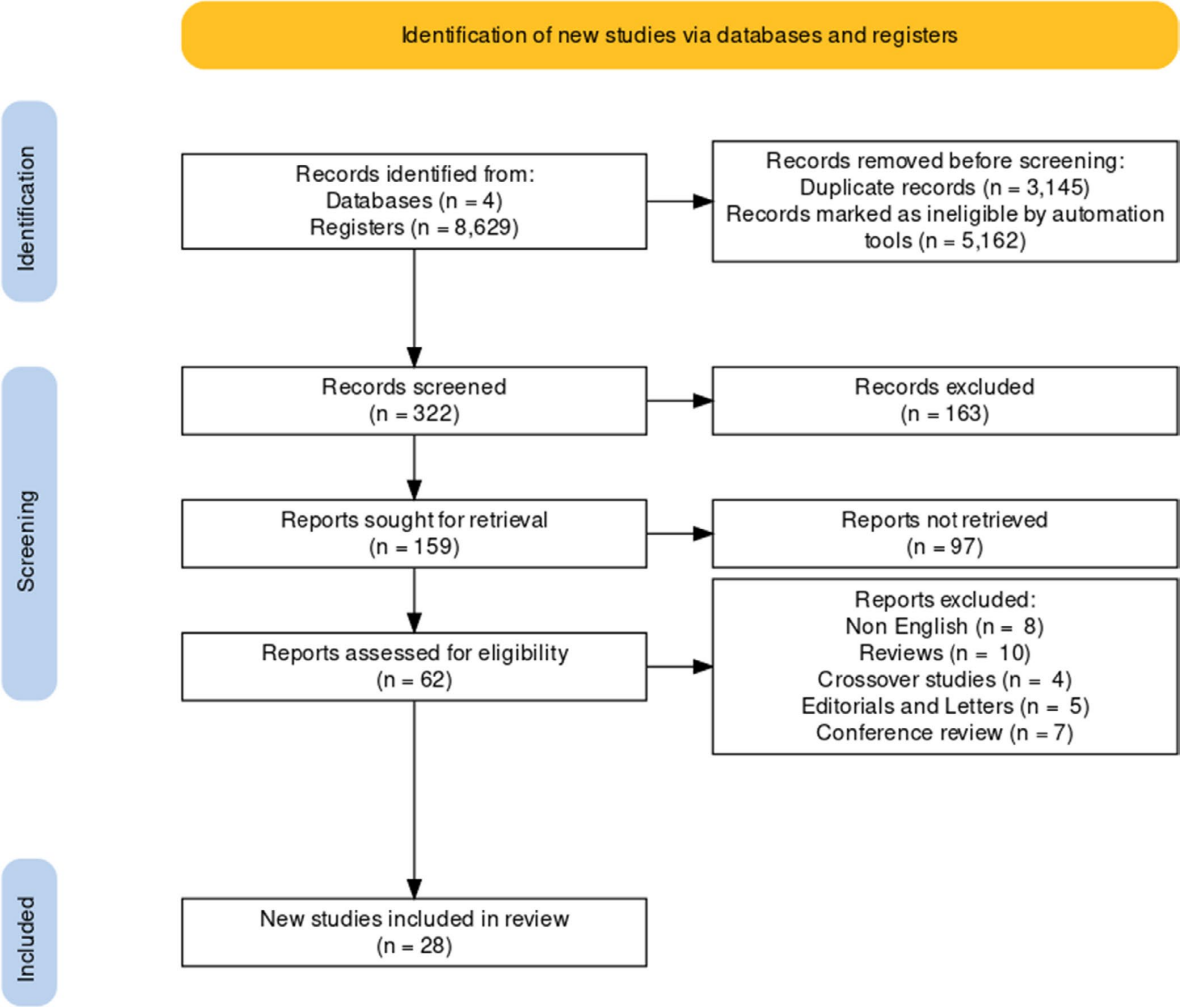
Flow diagram of literature search

RCTs were included if they were well‐described, had high quality, and defined outcomes, investigating the effects of probiotics on pregnant women. Non‐English articles, nonhuman trials, nonfull text studies, duplicate reports, and trials with insufficient data were excluded from the study. Figure 1 summarizes the search strategy.

### 2.4 Data extraction and quality assessment

Data were screened and analyzed independently by two authors, and any discrepancies were discussed to obtain consensus. Reference lists of all the related publications were also investigated to find any ignored articles. The publications cited in more than one database were included only once. A third researcher checked the results to ensure that all the eligible articles were evaluated. The initial phase of article selection consisted of the analysis of titles, abstracts, and finally reading the studies to select them based on the eligibility criteria. The information extracted from each study included the first author's last name, country of investigation, sample size (intervention/control), mean age and mean weigh of participants, study design, participants characteristics, intervention (probiotics), probiotics species, intervention dose, period of intervention, duration of following up, and outcome. The quality of the references was evaluated using the Joanna Briggs Institute (JBI; The Joanna Briggs Institute, 2014).^18^ RCTs were used to perform the quality assessment. Each component was rated as “yes,” “no,” “unclear,” or “not applicable.” A score ranging from 0 to13 points was attributed to each study. Ultimately, the studies with high quality were included in the present meta‐analysis. The File S3 shows JBI quality assessment.

### 2.5 Data analysis

Publication bias (Small study effect) was evaluated using Egger's linear regression test.^19^ To mean differences were estimated to compare the outcomes between intervention and control group, and a random‐effects model was used to pool results. The statistical analyses were performed using STATA software, version 16.0 (STATA Corporation, College Station, Texas, USA). Heterogeneity between studies was assessed by a Chi squared test and *I* ^2^ statistic. All the statistical interpretations were reported on a 95% confidence interval (CI) basis. *p*‐values less than 0.05 were considered as statistically significant.

## 3 RESULT

### 3.1 Search results

A total of 8629 articles were collected by searching the four electronic databases, among which 3145 were excluded due to duplication. After title, abstract, and full text assessment, 28 publications were retained for meta‐analysis (Figure 1).

### 3.2 Characteristics of the included studies

**FIGURE 2:**
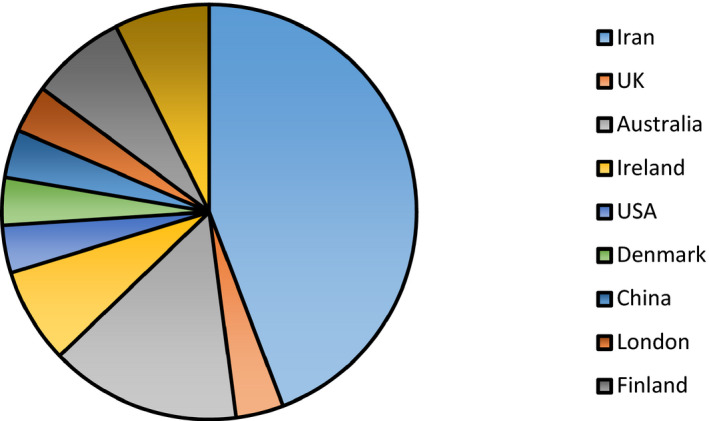
Frequency of studies on gestational diabetes among different countries

The methodological quality of the included studies was high for the RCT studies. (File S3). The age range of the pregnant women undergone by probiotics treatment was 18–40. Other factors regarding the included articles are shown in Table 1. According to Figure 2, most studies on GD were performed in Iran (7 out of the 28 studies and 909 out of the 4865 patients), followed by Australia, respectively.

**TABLE 1:** Characteristics of pregnant women included in all the 28 included studies

Condition	Prevalence (%) among studies	
Chronic disease	3%	
Smoking	11.1%	
Alcohol consumption	3%	
High BMI	59.25%	
Others	Activity	14.81%
	Mean SBP (mm Hg)	113.56 mm Hg
	Mean DBP (mm Hg)	71.715 mm Hg

Abbreviations: BMI, body mass index; DBP, diastolic blood pressure; SBP, systolic blood pressure.

Blood was the most prevalent specimen obtained from pregnant women in the articles. As shown in Table 2, among the 28 clinical trials included, 25 (88%) evaluated the effect of probiotics on GD, while 3 (11%) examined the synbiotics effects.

**TABLE 2:** The outcomes of different clinical trials assessing the probiotics efficacy on gestational diabetes among pregnant women

First author year	Origin	Sample size T C	Mean age (SD)	Mean weight (g) (SD)	Study design	Time of intervention	Probiotics	Probiotics Dose (**CFU)**	Duration of intervention	Controls used and duration of therapy	Outcomes	Certainty of the evidence (GRADE)
Allen, 2010	UK	454, 220 T 234 C	29 ± 5.6	NR	RCT DB prospective	36 wks of gestation	*L. salivarius CUL61*	6.25 × 10^9^	D 4wks	Placebo group Mothers during the last month of pregnancy Infants during the first 6 MO of life	The safe use of this consortium of organisms was suggested during pregnancy and early infancy	⊕ ⊕ ⊕ ⊕
							*L. paracaseiCUL08/*	1.25 × 10^9^				
							*B. animalis subsp. lactisCUL34*	1.25 × 10^9^				
							*B. bifidum CUL20*	1.25 × 10^9^				
Jafarnejad, 2016	Iran	82, 41 T 41 C	32.4 ± 3.1	70.4 ± 7.3	RCT	GDM	VSL#3 *lactic acid bacteria:* *S. thermophilus,* *Bifidobacterium* *breve,* *B. longum,* *B. infantis,* *L*. *acidophilus,* *L. plantarum* *L*. *Paracasei* *L. delbrueckii subsp. Bulgaricus*	112.5 × 10^9^	27–36 wks of gestation	Placebo group, 9 wks	1. Supplementation with probiotics may help modulate some inflammatory markers and may have benefits on glycemic control. 2. There were significant increase/decrease? in insulin levels and HOMA IR and a significant decrease in levels of IL−6 and hs‐CRP following probiotic consumption	⊕ ⊕ ⊕ ⊕
Mehri Jamilian, 2018	Iran	87, D + probiotic n = 30 Probiotic = 2 placebo n = 28	31.2 ± 5.9	71.7 ± 12.4	RCT DB	24–28 wks of gestation	*L. acidophilus,* *B. bifidum,* *L. reuteri* *L. fermentum*	8 × 10^9^ (each 2 × 10^9^)	D 6 wks	Not specified 24–28 wks of gestation	1. Vitamin D and probiotics resulted in a significant reduction in the levels of TG, VLDL, hsCRP, MDA, and HDL‐total cholesterol ratio 2. A significant rise in the levels of HDL‐cholesterol, total antioxidant capacity, TAC and total GSH	⊕ ⊕ ⊕ ⊕
Shahnaz Ahmadi, 2016	Iran	70, 35 T 35 C	28·5	77·7	RCT DB	24–28 wks of gestation	Symbiotic: *L. acidophilus,* *L. casei,B.ium bifidum*	2 × 10^9^ each	D 6 wks	Not specified 6 wks	1. Taking synbiotic supplements among patients with GDM had beneficial effects 2. Significant decrease in serum insulin levels and serum TAG and VLDL‐cholesterol concentrations	⊕ ⊕ ⊕ ⊕
Z Asemi, 2012	Iran	70, 37 T 33 C	24.2 ± 3.3	Not report	RCT DB	6–9 MO of pregnancy	*S. thermophilus,* *L. bulgaricus,* *L*. *acidophilus LA5,* *B. animalis BB12*	1 × 10^7^	D 9wks	Not specified 9 wks	1. Consumption of probiotic yogurt maintains serum insulin levels and HOMA‐IR score, which might help pregnant women prevent developing insulin resistance	⊕ ⊕ ⊕ 〇
Bita Badehnoosh, 2017	Iran	60, 30T 30C	28.8 ± 5.4	74.2 ± 9.5	RCT DB SC	24–28 wks of gestation	*L. acidophilus* *L. casei* *B. bifidum*	2 × 10^9^ each	D 6 wks	Not specified 6 wks	Significant decreases in the FPG, hs‐CRP levels and MDA/TAC ratio, as well as a significant increase in TAC level	⊕ ⊕ ⊕ 〇
Leonie K. Callaway, 2019	Australia	411, 207 T 204 C	31.3 ± 4.7	169	RCT DB	Second trimester pregnancy	*L. rhamnosus,* *B.animalis subsps lactis [BB−12]*	1 × 10^9^	D	Not specified 1 MO	Probiotics did not prevent GDM among overweight and obese pregnant women.	⊕ ⊕ 〇 〇
Neda Dolatkhah, 2015	Iran	64, 29 T 27 C	28.14 ± 6.24	83.27 ± 12.06	RCT DB	24–28wks of gestation	*L. acidophilus LA−5,* *B*. *BB−12* *S. thermophilus STY−31,* *L*. *delbrueckii bulgaricus LBY−27*	4 biocap>4 × 10^9^	8 wks	Not specified 8 wks	1. The probiotic supplement appeared to affect glucose metabolism and weight gain among pregnant women with GDM. 2. A decrease in the FBS level and insulin resistance index and an increase in insulin sensitivity index following probiotic consumption	⊕ ⊕ ⊕ 〇
Majid Hajifaraji, 2017	Iran	64, 29 T 27 C	28.1 ± 6.25	83.3 ± 12.1	RCT DB	24–28 wks of gestation	*L. acidophilus* *LA−5,* *B. BB−12,* *S. Thermophilus STY−31,* *L. delbrueckii bulgaricus LBY−27*	4 biocap>4 × 10^9^	D 8 wks	Not specified 8 wks	1. The probiotic supplement improved several inflammation and oxidative stress biomarkers in women with GDM 2. Sh‐ CRP, TNF‐α, malondialdehyde, glutathione reductase, and erythrocyte glutathione peroxidase levels were improved, while serum IL−6 levels was decreased	⊕ ⊕ ⊕ ⊕
Maryam Karamali, 2017	Iran	60, 30 T 30 C	27.2 ± 5.9	74.7 ± 10.5	RCT DB	NR	Symbiotic: *L*. *acidophilus strain T16(IBRCM10785)*, *L. casei strain T2 (IBRC‐M10783),* *B*. *bifidum strain T1 (IBRC*‐*M10771*)	2 × 10^9^	6 wks	Not specified 6 wks	Probiotic consumption increased serum hs‐CRP, plasma malondialdehyde, cesarean section rate, and incidence of hyperbilirubinemic newborns while decreased the levels of TAC and GSH	⊕ ⊕ ⊕ 〇
Athasit Kijmanawat, 2019	USA	57, 28 T 29 C	32.50 ± 5.02	63.49 ± 10.75	RCT DB	6–7 MO of gestation	*Bifidobacterium*	10^9^	D 4 wks	Not specified 4 wks	Probiotic consumption increased the fasting plasma glucose, fasting plasma insulin, insulin sensitivity, and homeostatic model assessment for insulin resistance and decreased fasting glucose	⊕ ⊕ ⊕ 〇
							*Lactobacillus*	10^9^				
Karen L. Lindsay, 2015	Ireland	149, 74 T 75 C	>18 y	33.5 ± 5.0	RCT DB	<34 wks gestation	*L. salivarius UCC118*	10^9^	D 4 wks	Not specified 4 wks	Probiotic consumption had no impacts on glycemic control	⊕ ⊕ ⊕ 〇 ⊕ ⊕ ⊕ 〇
Zohoor Nabhani, 2018	Iran	90, 45 T 45 C	Synbiotic 29.4 ± 5.8	69 ± 12.8	RCT DB	24–28 wks of gestation	*L.acidophilus*	5 × 10^10^	6 wks	Not specified 6 wks	1. Probiotic consumption may prevent any increments in LDL‐C levels as well as having positive effects on HDL‐C and TAC status. 2. Positive effect of synbiotics on SBP and DBP was noticeable.	
							*L.plantarum*	1.5 × 10^10^				
							*L.fermentum*	7 × 10^9^				
							*L. Gasseri*	2 × 10^10^				
Marloes Dekker Nitert1, 2013	Australia	540, 270 T 270 C	>18.0	NR	RCT DB MC prospective	GDM at 28 wks gestation	*L.rhamnosus GG,* *B. lactis BB−12*	1 × 10^9^ each	D	Not specified > 2 y	Probiotics prevented gestational diabetes in the high‐risk group of overweight and obese pregnant women.	⊕ ⊕ ⊕ ⊕
Outi Pellonperä, 2019	Finland	439, Probiotic n = 109 probiotic/fish oil n = 110 fish oil/placebo n = 109 placebo/placebo n = 110	Fish oil + probiotics 30.8 ± 4.6	83.6 ± 14.9	RCT DB	mean 13.9 ± 2.1 gestational wks	*Lactobacillus rhamnosus* *HN001,* *Bifidobacterium animalis ssp. lactis 420*	10^10^ each	D	Throughout the pregnancy, until 6 MO postpartum.	Intervention with fish oil and probiotics did not lower the incidence of GDM, fasting glucose concentration, or insulin resistance in overweight and obese pregnant women	⊕ ⊕ ⊕ 〇
Kristin L. Wickens, 2017	New Zealand	423, 212 T 211 C	30 36	63–80	RCT DB TC	Earliest first‐trimester, 14–16 wks of gestation	*L. rhamnosus HN001*	6 × 10^10^	D	Throughout pregnancy until 6 MO post birth if still breast‐feeding	Probiotics may reduce GDM prevalence particularly among older women and those with previous GDM.	⊕ ⊕ ⊕ 〇
Hanieh Asgharian, 2019	Iran	130, 65 T 65 C	29.5 ± 6.2	Birth weight (g) 3270 ± 495	RCT	BMI ≥25, FPG<92 mg/dl, 22 wks of gestation	*L. acidophilus La5,* *B. lactis Bb12*	5 × 10^8^ each	D 12wks	Until 1 MO after birth	The probiotics supplementation had some beneficial effects on glucose metabolism of overweight and obese pregnant women	⊕ ⊕ ⊕ ⊕
Mahtab Babadi, 2018	Iran	48, 24 T 24 C	28.8 ± 4.3	70.1 ± 5.2 kg	RCT DB PCCT	GDM at 24–28 wks of gestation	*L. acidophilus,* *L. casei,* *B. bifidum,* *L*. *fermentum*	2 × 10^9^ each	D 6 wks	1 y	1. Probiotic had beneficial effects on gene expressions related to insulin, inflammation, and glycemic control 2. Probiotics decreased lipid profiles, inflammatory markers, and oxidative stress	⊕ ⊕ ⊕ ⊕
Christine Barthow, 2016	New Zealand	400	NR	NR	RCT DB TC	14–16 wks of gestation	*L. rhamnosus HN001*	6 × 10^9^	D	12–16 wks, until 6 MO post‐partum	Probiotics alleviated the severity of eczema and atopic sensitisation in the first year of life of neonates.	⊕ ⊕ ⊕ ⊕
Luisa F. Go mez‐Arango, 2016	Australia	205	Overweight 36^33^, ^34^, ^35^, ^36^, ^37^, ^38^ Obese 36^32^, ^33^, ^34^, ^35^, ^36^, ^37^, ^38^, ^39^, ^40^	BMI: Overweight 27.5 (26.4–28.4) Obese 34.9 (32.1–38.5)	RCT	16 wks of gestation	*L. rhamnosus GG* *B. lactis BB−12*	2 × 10^9^ each	NR	NR	1. The abundance of butyrate‐producing bacteria in the gut microbiota was negatively associated with BP and with PAI−1 levels. 2. Increasing butyrate‐producing capacity may contribute to maintenance of normal BP in obese pregnant women	⊕ ⊕ 〇 〇
Luisa F. Gomez‐Arango, 2017	Australia	57 overweight 73 obese	Overweight: 32.0 (29.0 –34.0) Obese: 30.5 (28.0–34.0)	BMI (kg/m) Overweight: 27.9 (27.0 –29.1) Obese 34.3 (31.8–41.3)	RCT	16 wks of gestation	*L. rhamnosus GG* *B. lactis BB−12*	2 × 10^9^	D	1–16 wks gestation	1. Low dietary fiber may enable overgrowth of *Collinsella* spp.and alter the overall fermentation pattern in the gut microbiota 2. That dietary choices during pregnancy can modify the nutritional ecology of the gut microbiota, with potential deleterious effects on the metabolic and inflammatory health of the host.	⊕ ⊕ 〇 〇
Sofie Ingdam Halkjaer, 2016	Denmark	50, 25 T 25 C	> 18 y	BMI of between 30–35 kg/m2	DB SC RPCT	14–20 wks of gestation	*S. thermophilus DSM 24731,* *B. breve DSM 24732,* *B*. *longum DSM 24736,* *B. infantis* *DSM 24737,* *L. acidophilus* *DSM 24735,* *L. plantarum DSM 24730,* *L. paracasei DSM 24733,* *L. delbrueckii,* *bulgaricus DSM 24734)*	450 billion each	12–16 wks	12–16 wks infants until 9 MO	1. Probiotics could control weight gain and reduce complications during pregnancy by inducing changes in the gut microbiota 2. Probiotics could influence the infant's microbiota, which could have important implications on infant's development and health	⊕ ⊕ ⊕ ⊕
Karen L Lindsay, 2014	Ireland	138, 63 T 75 C	31.4 ± 5.0	89.5 ± 9.1	DB RPCT	24–28 wks of gestation	*L. salivarius UCC118*	10^9^	D 4wks	<20 wks of gestation	Probiotics did not influence the maternal fasting glucose, the metabolic profile, or pregnancy outcomes in obese women	⊕ ⊕ ⊕ 〇
Raakel Luoto, 2012	Finland	256, Diet/probiotics n = 64 Diet/placebo (n = 59) Placebo/control (n = 58)	29.7 (4.3)	Infants (g) 3468 (3360–3577)	RPCT Prospective	every trimester of pregnancy infant age of 6 MO	*L. rhamnosus GG,* *B. lactis*	10^10^ each	D every trimester of pregnancy	2002–2005	The dietary intervention increased the colostrum adiponectin concentration	⊕ ⊕ ⊕ 〇
Farnaz Sahhaf Ebrahimi, 2019	Iran	84, 42 T 42 C	31.64 ± 5.97	79.5 ± 17.31	DB RPCT	3–6 MO	*L. acidophilus* *B. lactis*	300 g/day 10^6^	D 8 wks	2 MO	Probiotics increased fasting and post prandial blood glucose and decreased the level of HbA1c, in lower weight and fewer macrosome neonates	⊕ ⊕ ⊕ ⊕
Lihui Si, 2019	China	226 113+113	34.32 ± 6.47	58.48 ± 7.36	Parallel RCT	12.14 ± 2.46 wk of gestation	*L. bulgaricus*	10^8^	7 d	40 wks	*L*. *bulgaricus* improved the antioxidant capacity of black garlic in the prevention of GDM	⊕ ⊕ ⊕ ⊕
Shaun Sabico, 2017	UK	78, 39 T 39 C	48.0 ± 8.3	75.6 ± 11.0 kg	RCT DB SC	T2DM patients	*B. bifidum W23,* *B.lactis W52,* *L. acidophilus W37,* *L*. *brevis W63,* *L. casei W56,* *L. salivarius W24,* *L. lactis W19,* *L. lactis W58*	2.5 × 10^9^ each	12 wks	12/13 wks	Probiotics significantly improved HOMA‐IR and modestly reduced abdominal adiposity among medication naïve T2DM patients	⊕ ⊕ ⊕ ⊕
Maryam Karamali, 2018	Iran	60 30 T 30 C	P: 27.2 ± 4.6 C: 27.7 ± 4.7	62.9 ± 7.8 63.7 ± 8.0	RCT DB	women with PCOS	*L. acidophilus, L. casei and B. bifidum*	2 × 10^9^ each	12 wks	12 wks	Probiotic supplementation of PCOS had beneficial effects on total testosterone, SHBG, mFG scores, hs‐CRP, TAC, and MDA levels but did not affect other metabolic profiles.	⊕ ⊕ ⊕ ⊕

Abbreviations: BP, blood pressure; C, control; CFU, colony‐forming units; Chol, cholesterol; C‐peptide, connecting peptide; D, daily; d, days; DB, double‐blind; FBS, fasting blood sugar; GDM, gestational diabetes mellitus; GSH, total glutathione; HDL‐cholesterol, high‐density lipoprotein‐cholesterol; HOMA‐IR, homeostatic model assessment of insulin resistance; HOMA‐β, homeostasis model assessment of β‐cell function; hs‐CRP, high‐sensitivity C‐reactive protein; IL, interleukin; INS, insulin; LDL‐cholesterol, low‐density lipoprotein‐cholesterol; MC, multi‐center; MDA, malondialdehyde; mF‐G, modified Ferriman‐Gallwey; MO, month; NO, nitric oxide; NR, not report; PAI‐1, plasminogen activator inhibitor‐1; PCCT, placebo‐controlled clinical trial; PCOS, Polycystic ovary syndrome; QUICKI, quantitative insulin sensitivity check index; RCT, randomized controlled trials; RPCT, randomized placebo‐controlled study; SC, single‐center; SHBG, sex hormone‐binding globulin; T, test; T2DM, Type 2 diabetes mellitus; TAC, total antioxidant capacity; TC, two‐center; TG, triglyceride; TNF‐α, tumor necrosis factor alpha; VLDL‐cholesterol, very low‐density lipoprotein‐cholesterol; wks, weak(s); y, year.

**FIGURE 3:**
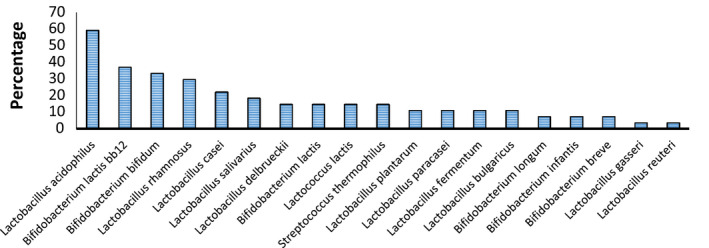
Frequency of different types of probiotic species used in different studies

Generally, among a total of 19 different species used as probiotics in the studies (Figure 3), *Lactobacillus acidophilus* (59.25%) and *Bifidobacterium lactis* Bb12 (37.03%) were the two most widely used probiotic species. The mean daily dosage of probiotics used for the treatment of GD among different studies was determined as 4.63 × 10^7^ colony forming units (CFU), with a minimum and maximum range of 10^5^–4.5 × 10^14^ CFU, respectively. This probiotic dosage totally lasted 4 to 40 weeks and in a few cases 9 month after delivery. Five studies (18.51%) used only one strain as the probiotic treatment, while 22 out of the 28 clinical trials used a mixture of probiotic bacteria (81.48%), in a way that 9 articles used two species, 3 articles used 3 species, 3 articles used 8 different species, and 7 articles used 4 bacterial species (only one trial examined four different *Lactobacillus* species for GD treatment among pregnant women). In the current meta‐analysis, the effect of probiotics/synbiotics on GD has been assessed through different measures, categorized as primary and secondary according to previous articles.^9^, ^20^ Primary measures were defined as metabolic or biochemical factors including fasting glucose, glycated hemoglobin (HbA1c) level, serum insulin, quantitative insulin sensitivity check index (QUICKI), Homeostatic Model Assessment for Insulin Resistance (HOMA‐IR). Secondary measures were further classified as mother‐ or infant‐related. Mother‐related secondary measures (also known as maternal measures) included changes in the lipid profiles, inflammatory markers and oxidative stress, preeclampsia, gestational hypertension, hypertensive disorders of pregnancy, incidence of caesarean delivery, excess weight gain, and change in the prevalence of probiotic bacteria in the gut microbiome. Infant‐related secondary measures (or neonatal measures) included bone fracture, stillbirth or neonatal death, gestational age at delivery (weeks), the incidence of macrosomia, the incidence of preterm delivery, the incidence of newborns’ hyperbilirubinemia, and the incidence of newborns’ hypoglycemia.^20^

### 3.3 Effects of probiotic supplementation on the metabolic status of pregnant women

Among the 28 studies included in the current meta‐analysis, only 16 contained meta‐analysis able data on the effect of probiotics (or synbiotics) supplementation on metabolic (biochemical) parameters. The data are gathered in Table 2. In all these studies, blood samples were collected from volunteers following probiotics/synbiotics supplementation, at the beginning and the desired week of the trial after which the analysis of metabolic parameters were carried out according to the specified protocols. Metabolic parameters in these studies can be classified in three distinct groups; glycemic status, lipid profiles and inflammatory markers and oxidative stress. These biochemical parameters are regarded in this meta‐analysis as primary measures, which should not be confused with the maternal (during pregnancy or and postpartum) or neonatal secondary measures.

#### 3.3.1 Glycemic status

**FIGURE 4:**
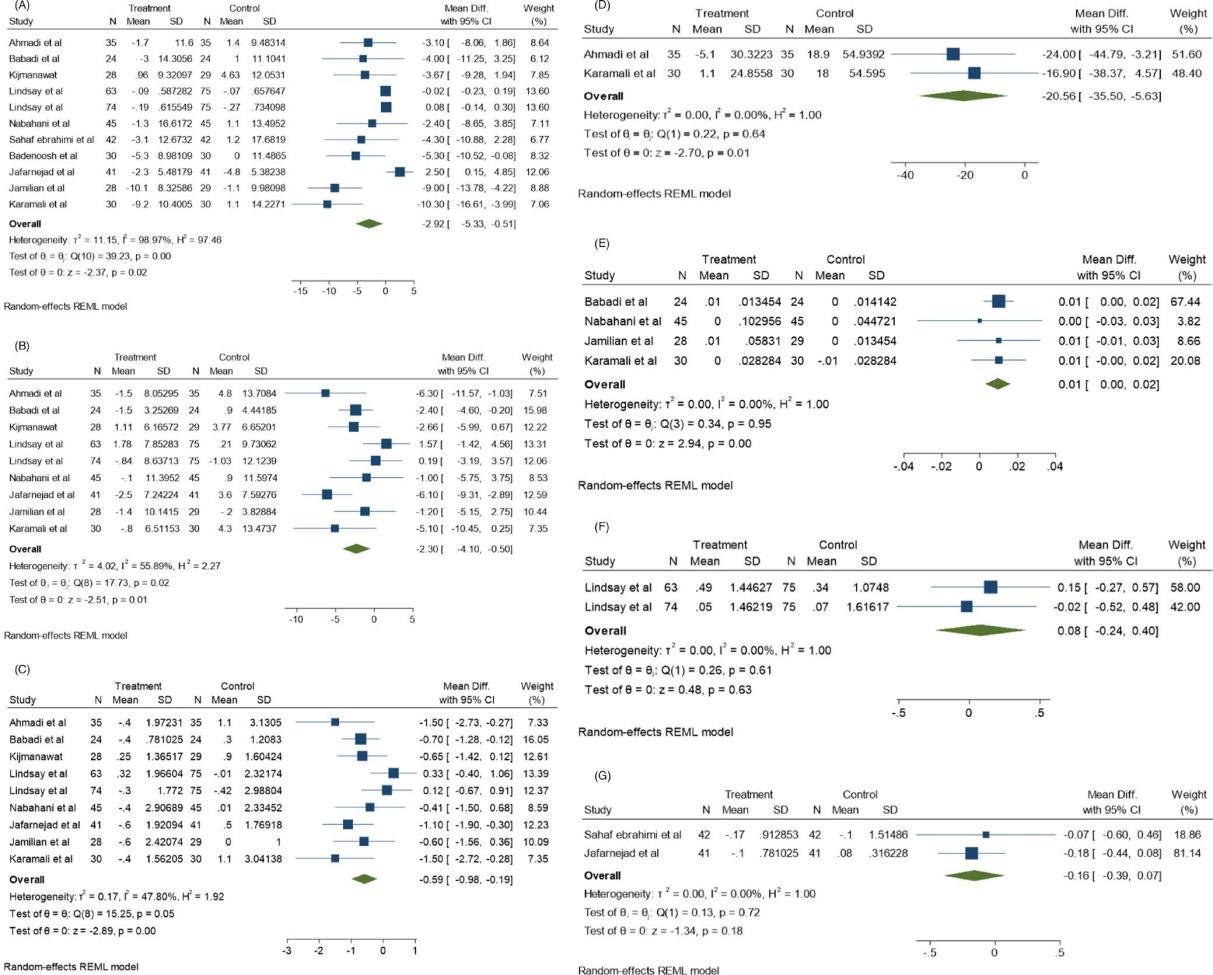
Effect of probiotics supplementation on glucose status (A) FBS levels, (B) Insulin levels, (C) HOMA‐IR, (D) HOMA‐B, (E) QUIKI, (F) C‐peptide, and (G) HbA1c in pregnant women

Glycemic status was evaluated through different criteria including FBS, INS, HOMA‐IR, HOMA‐B, QUICKI, C‐peptide, and HbA1c levels. Among the articles examining FBS levels before and after probiotics administration, 11 were significantly correlated with heterogeneity (*I* ^2^ = 98.97%, *p* = 0.00). As shown in Figure 4A, the mean difference (MD) of FBS shows a significant decrease compared to the control group (*p* < 0.05). Also, according to Figure 4B‐G, probiotic supplementation was able to decrease INS, HOMA‐IR, HOMA‐B, QUICKI, C‐peptide (*p* < 0.05), and HbA1c (*p* > 0.05). Moreover, INS (*I* ^2^ = 55.89%, *p* = 0.00) and HOMA‐IR (I^2^ = 47.80%, *p* = 0.05) values showed significant heterogeneity among 9 studies of this meta‐analysis, while HOMA‐B (*I* ^2^ = 0.00%, *p* = 0.64), C‐peptide (*I* ^2^ = 0.00%, *p* = 0.61), and HbA1c (*I* ^2^ = 0.00%, *p* = 0.72) among two studies and QUIKI (*I* ^2^ = 0.00%, *p* = 0.95) among four studies did not show a significant heterogeneity, respectively. It is worth being noted that Jamilian et al. evaluated the synergistic effects of 50,000 IU vitamin D3 and probiotic on metabolic status of three different groups^21^ and Luoto et al. determined colostrum adiponectin concentration in pregnant women after consumption of probiotics.^22^ These studies showed that the maternal diet, as well as vitamin D3 and probiotics co‐supplementary, are highly effective on metabolic factors and colostrum adiponectin concentration causing metabolic hemostasis in pregnant woman. Probiotic bacteria are able to increase the antioxidant function of some natural compounds. For example, Li et al.’s study showed that *L*. *bulgaricus* is able to increase the ability of black garlic to scavenge toxic radicals, besides being able to reduce FBG levels. Therefore, probiotics have synergistic effects with black garlic to improve the symptoms of GD.^23^

**FIGURE 5:**
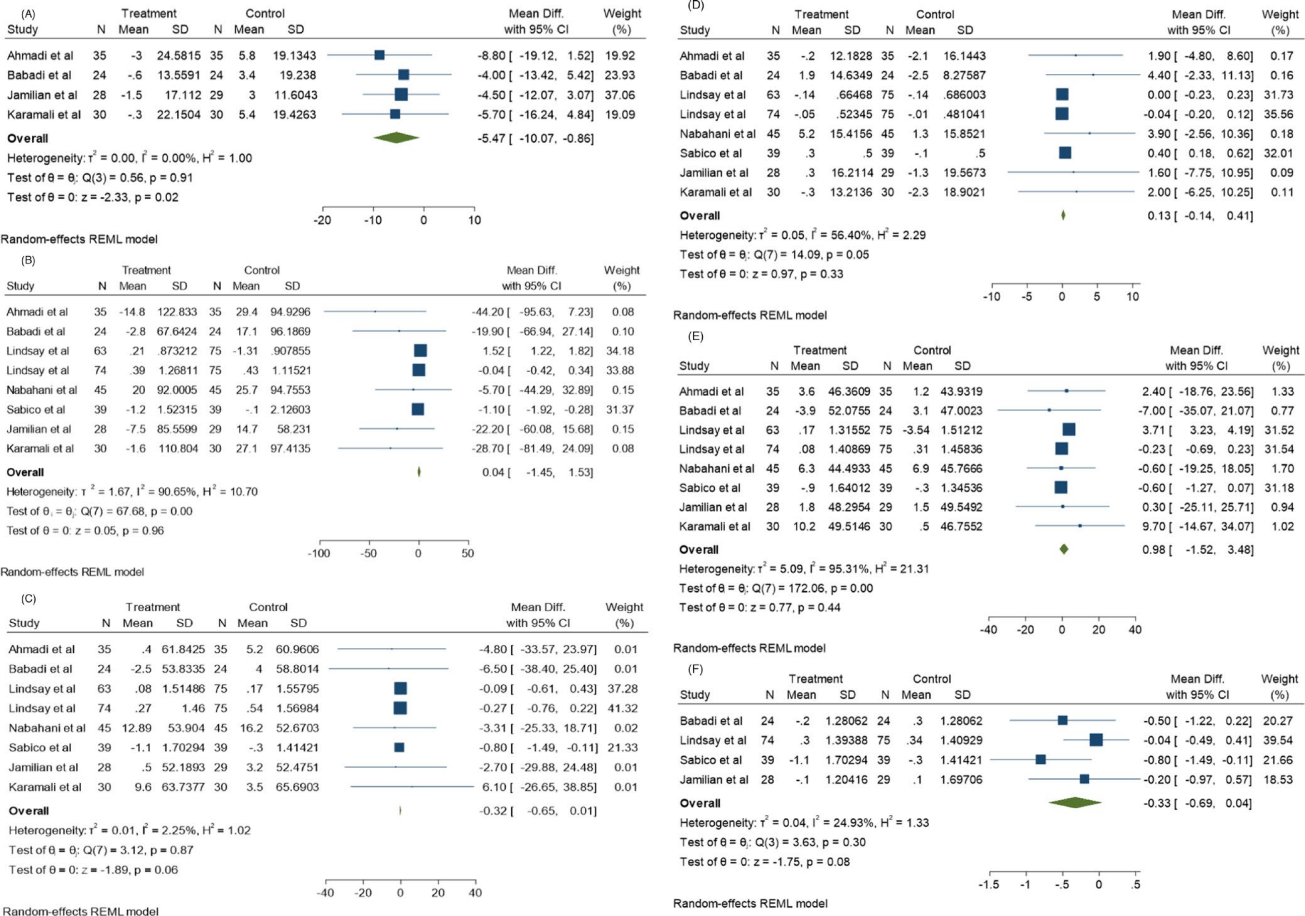
Effect of probiotics supplementation on lipid profiles (A) VLDL levels, (B) TG, (C) Chol, (D) HDL, (E) LDL, (F) Total HDL/Chol ratio in pregnant women

#### 3.3.2 Lipid profiles

Serum total cholesterol/HDL ratio (Chol/ HDL), Low‐density lipoprotein (LDL), High‐density lipoprotein (HDL), triglycerides, total cholesterol/HDL ratio, very low‐density lipoprotein (VLDL) and cholesterol: HDL ratio have been considered as lipid profile indicators in different investigations. Consumption of probiotics has been shown to significantly decrease the mean VLDL level (*p* < 0.05). As shown in Figure 5A, no significant heterogeneity correlation (*I* ^2^ = 0.00%, *p* = 0.91) exists among the 4 studies evaluating VLDL before and after probiotic consumption. According to Figure 5B–F, no significant differences were found in the mean TG, Chol, HDL, and levels in 8 different studies assessing these factors and the total Chol/HDL ratio among the 4 corresponding studies (*p* > 0.05). However, significant heterogeneity was found in the TG (*I* ^2^ = 90.65%, *p* = 0.00), HDL (*I* ^2^ = 56.40%, *p* = 0.05), and LDL (*I* ^2^ = 95.31%, *p* = 0.00) levels among 8 studies, as well as Chol (*I* ^2^ = 2.25%, *p* = 0.87) levels among 8 studies. On the other hand, the Chol/HDL ratio (*I* ^2^ = 0.00%, *p* = 0.91) represented no heterogeneity among the 4 corresponding studies.

#### 3.3.3 Inflammatory markers and oxidative stress

**FIGURE 6:**
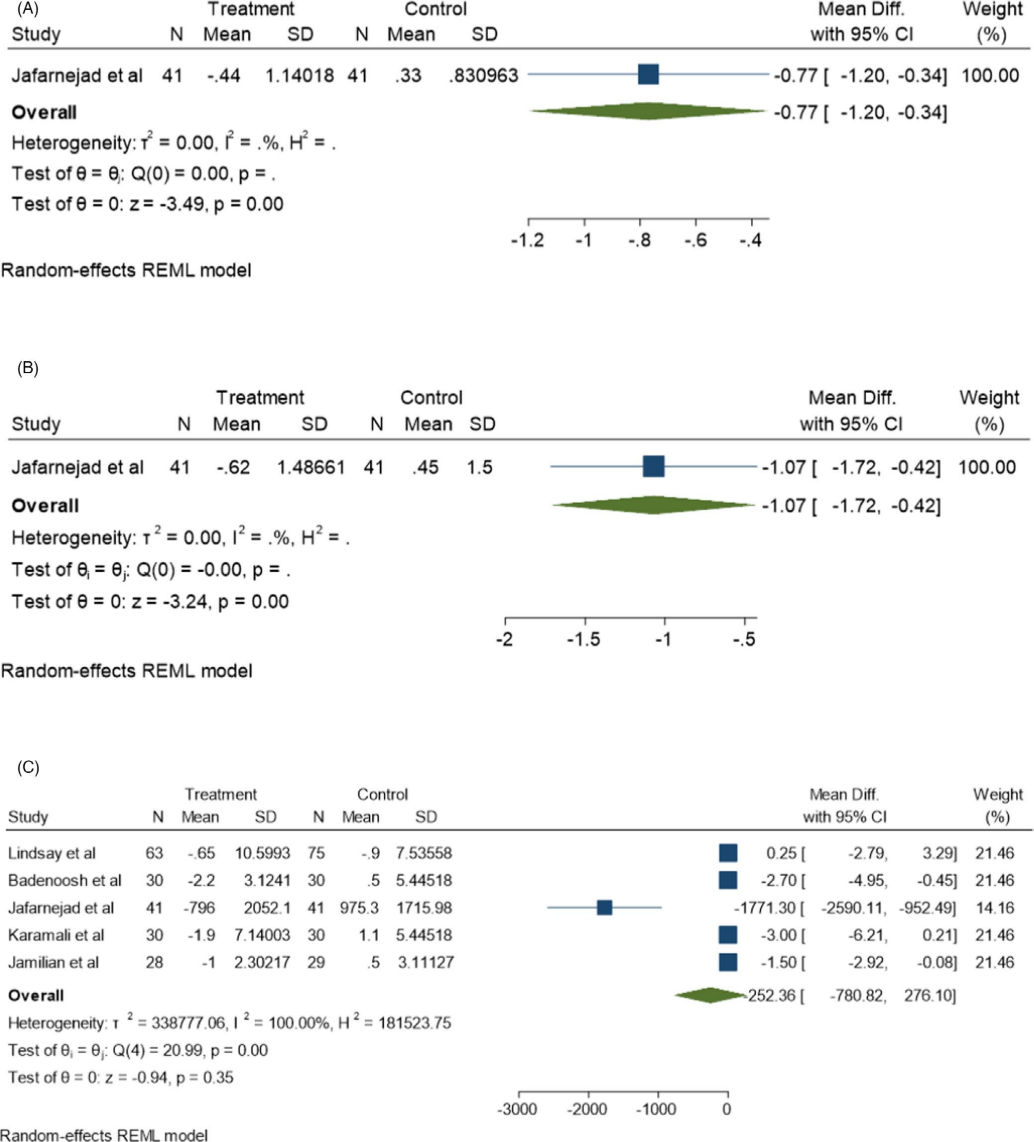
Effect of probiotics supplementation on inflammatory markers (A) IL‐6, (B) TNF‐α and, (C) CRP in pregnant women

Inflammatory markers and oxidative stress represent inflammatory conditions in the body which have been evaluated in some studies by assessing the levels of IL‐6, TNFα, CRP, malondialdehyde (MDA), total antioxidant capacity (TAC), glutathione (GSH), NO, and adipocytokines such as leptin, adiponectin, and resistin. In this meta‐analysis, the post‐probiotic levels of IL‐6 (Figure 6A) and TNF‐α (Figure 6B) were evaluated only in Jafarnejad et al.’s study,^24^ which showed a significant change after probiotic consumption (*p* < 0.05).

**FIGURE 7:**
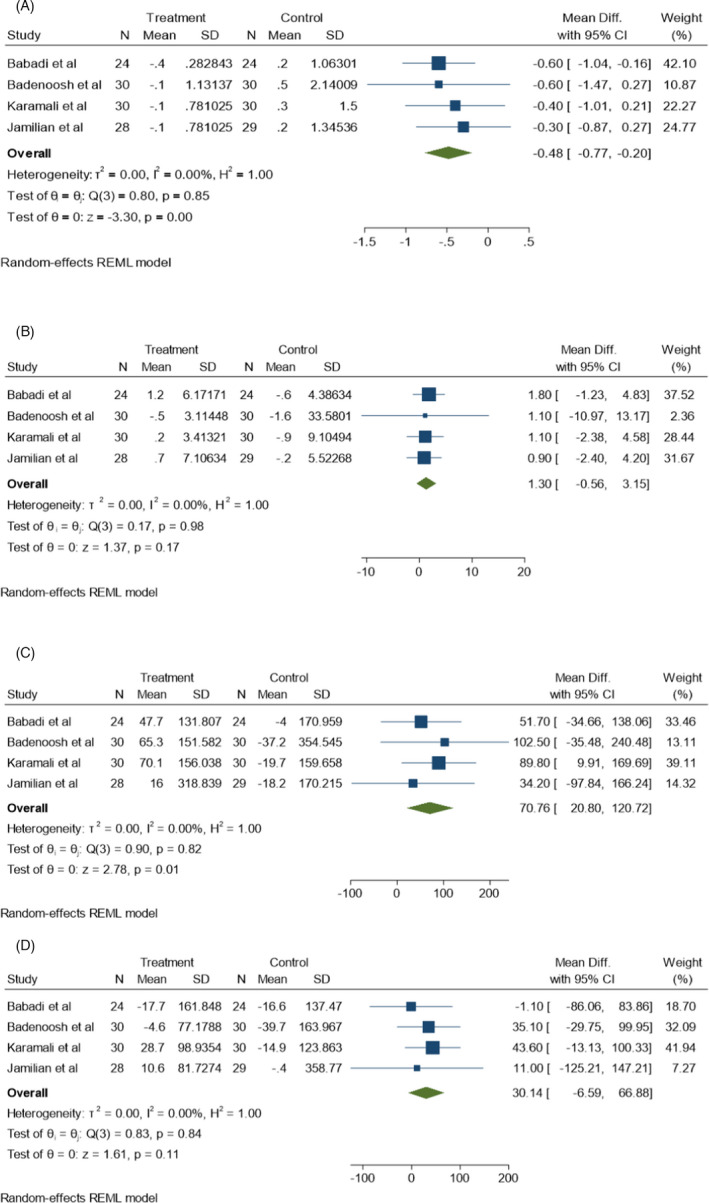
Effect of probiotics supplementation on oxidative stress markers (A) MDA, (B) NO, (C) TAC and, (D) GSH in pregnant women

MDA (*I* ^2^ = 0.00%, *p* = 0.85), NO (*I* ^2^ = 0.00%, *p* = 0.98), TAC (*I* ^2^ = 0.00%, *p* = 0.82), and GSH (*I* ^2^ = 0.00%, *p* = 0.84) levels were assessed in 4 different studies which had no significant heterogeneity correlation among them. No significant increase was found in the mean levels of NO and GSH compared to the control groups (Figure 7B,C) (*p* > 0.05). The TAC level was significantly increased and the MDA level was significantly decreased following probiotic supplementation in these studies (Figure 7A,C) (*p* < 0.05). However, no significant reduction was found in the mean CRP level following probiotic consumption compared to the control group (*p* > 0.05). According to Figure 6C, there are significant heterogeneity correlations (*I* ^2^ = 100%, *p* = 0.00) among the 5 corresponding studies used in this meta‐analysis.

### 3.4 Colostrum adiponectin levels

Adiponectin, as a protein hormone, plays important roles in obesity‐associated diseases such as type 2 diabetes when present at low levels in serum.^25^ This molecule shows sensitivity to insulin and has anti‐inflammatory effects. Lueto et al. showed that intake of probiotic‐supplemented diets (combination of *Lactobacillus rhamnosus* GG and *Bifidobacterium lactis*) significantly increases the adiponectin concentration in breast milk of mothers compared to the placebo group (12.7 vs. 10.2 (*p* = 0.024)), and this can immunologically support the neonates.^22^

### 3.5 Microbiome, maternal and neonatal health

Among the studies included in this meta‐analysis, two examined the effect of probiotics on microbiome population, specifically intestinal microbiome, among pregnant women.^15^ Because the composition of the microbial flora changes during obesity and overweight, these changes can affect blood pressure and inflammation. For this purpose, the amount of plasminogen activator 1 inhibitor in obese pregnant women was measured in some studies. It has been shown that the number of butyrate‐producing bacteria in the intestinal microbiome is inversely related to the amount of plasminogen activator inhibitor 1. Thus, the presence of butyrate‐producing bacteria and consequently butyrate as their metabolic product is important in maintaining normal blood sugar in women during pregnancy. Halkjaer et al. investigated the effect of probiotics on maternal overweight, as the most significant side effects during pregnancy, as well as other side effects that affect both the mother and neonate. The results showed a higher incidence of diabetes and overweight among pregnant women due to changes in the composition of the gut microbiome. This indicates a direct correlation between the gut microbiome composition (which can effectively be regulated by probiotic supplementation) and the health status of the mother. Probiotic consumption can cause metabolism regulation in mother, leading to a reduction in GD incidence, which can, ultimately affect the health status of the neonate.^26^

### 3.6 Risk of bias assessment

**FIGURE 8:**
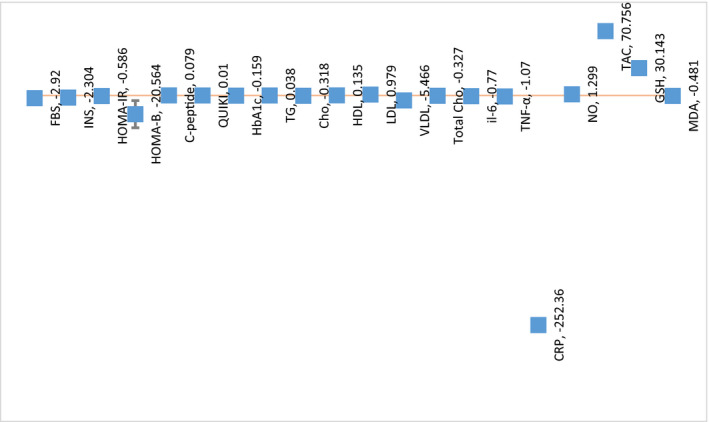
Mean differences of variables between probiotic and placebo groups among different studies

The mean differences and results of the Egger test are displayed in Figure 8 and Table 3. There was a publication bias in the meta‐analysis of the FBS, TG, and CRP groups (*p* < 0.05).

**TABLE 3:** Extracted data for meta‐analysis

	*I* ^2^*	p**	Mean differences	95% CI*** (LCI, HCI)	Egger test
FBS	98.97	0.00	−2.92	(−5.33, −0.51)	0.0042
INS	55.89	0.01	−2.304	(−4.11, −0.51)	0.3496
HOMA‐IR	47.80	0.00	−0.586	(−0.98, −0.18)	0.1597
HOMA‐B	0.00	0.01	−20.564	(−35.49, −5.63)	—
C‐peptide	0.00	0.63	0.079	(−0.24, −0.39)	—
QUIKI	0.00	0.00	0.01	(0.003,0.016)	0.6804
HbA1c	0.00	0.18	−0.159	(−0.4,0.07)	—
TG	90.65	0.96	0.038	(−1.5, 1.53)	0.0186
Chol	2.25	0.06	−0.318	(−0.65, 0.01)	0.6211
HDL	56.40	0.33	0.135	(−0.13, 0.40)	0.0942
LDL	95.31	0.44	0.979	(−1.5, 3.47)	0.9903
VLDL	0.00	0.02	−5.466	(−10.07, −0.86)	0.6576
Total chol/HDL ratio	24.93	0.08	−0.327	(−0.7, 0.03)	0.1815
IL−6	—	0.00	−0.77	(−1.20, −0.33)	—
TNF‐a	—	0.00	−1.07	(−1.71, −0.42)	—
CRP	100.00	0.35	−252.36	(−780.82, 276.10)	0.0001
NO	0.00	0.17	1.299	(−0.55, 3.15)	0.9365
TAC	0.00	0.01	70.756	(20.79, 120.71)	0.9033
GSH	0.00	0.11	30.143	(−6.59, 66.87)	0.5055
MDA	0.00	0.00	−0.481	(−0.76, −0.19)	0.8535

Note

HbA1c or Hemoglobin A1C: glycated hemoglobin, (* I2: Index of dispersion, **p value, *** confidence interval).

### 3.7 Level of evidence

The level of evidence based on GRADE was shown in Table 2. Certainty of results assessed in this meta‐analysis/systematic review “The Effect of Probiotics on Gestational Diabetes and its complications in pregnant mother and newborn” were considered as effective and represent low risk of bias. Almost all trials (twenty‐five) showed probiotics have improvement effect during pregnancy and early infancy, except for three trials which announced that probiotics has no effect on GD.^9^, ^27^, ^28^

## 4 DISCUSSION

Elevated levels of placental and pregnancy‐associated hormones or cytokines have the potential to increase the risk of GD. The use of safe and cost‐effective therapies is essential for the prevention and management of GD. Reports have shown that lifestyle intervention, including change in the diet and exercise, is the cornerstone for the prevention and treatment of GD.^29^ At present, several clinical trials have documented that regular consumption of probiotics effetely improves maternal metabolism and pregnancy outcomes.

This meta‐analysis revealed that taking probiotic supplements during pregnancy by women with GD has beneficial effects on the metabolic status, colostrum adiponectin levels, microbiome composition, and the maternal and infant health. However, 4 studies reported no significant effect for the probiotic intervention on the incidence of GD.^27^, ^28^, ^30^, ^31^ The difference in the results of these studies may be due to different studies included in the meta‐analyses, and also the different method of statistical analysis.

Probiotic supplements may contain either one strain of bacteria or a mixture of two or more strains/species. Studies have shown that the use of multi‐strain and/or multi‐species probiotics may in some cases be more effective than single‐strain probiotics because multiple strains/species may synergistically augment the effects of each other.^32^ The administration of synbiotics rather than probiotics seems to increase the overall beneficial outcome due to the synbiotics ability to improve the viability of the probiotic bacteria by supplying them with energy and nutrients. Among the studies investigated in this meta‐analysis, the two species *Lactobacillus acidophilus* and *Bifidobacterium lactis* Bb12 were widely used to treat women with GD and to better control their metabolic status through pregnancy. The 2 bacterial genera *Lactobacillus* and *Bifidobacterium* are the world's most commonly recorded probiotics that provide excellent therapeutic benefits in many clinical conditions.^33^, ^34^ They are also the most well‐characterized probiotic supplements in food industry,^35^ the genomic, biological, and physiological features of which have been investigated by many investigators. It is essential to assess the features, safety, and efficacy of the used probiotic strains in clinical trial studies. Probiotic dose is an important parameter to consider when examining the probiotics effects on the physiological functions in human and animals. FAO/WHO has proposed that adequate amounts of probiotics can bring health benefits to the host. Although the optimal probiotics dosage is not yet clear, it is generally accepted that a probiotic dose of >10^6^ CFU/g (CFU/mL) can render highly efficient outcome.^36^ In trials included in this study, women with GD received daily probiotic doses from 10^5^ to 10^14^ CFU during pregnancy or after deliver and an average dose of ≥10^7^ CFU/day was recommended for modulation of GD during pregnancy. These findings are consistent with the results of Han et al.’s meta‐analysis study.^37^ On the other hand, 4 clinical trials in this study showed that the dose of ≥10^7^ CFU/day had no beneficial influence on pregnancy outcomes such as maternal metabolic profiles and GD incidence.^9^, ^27^, ^28^, ^31^ This difference can be due to variations in the types of probiotic species/strains used, probiotics formulations, duration of interventions, and patients’ conditions. The changes in body homeostasis (through physiological changes including increase in maternal hormone levels and BMI) are associated with GD risk during pregnancy, which might be correlated with adverse pregnancy outcomes such as impaired glycaemia, macrosomia,^38^ pre‐eclampsia, preterm birth,^39^ and metabolic syndrome postpartum. Assessments of RCTs in this review showed probiotics/synbiotics supplementation have beneficial effects on the metabolism of insulin, lipids profile, biomarkers of inflammation, and oxidative stress. These supplements significantly reduced insulin resistance (FBS levels, serum insulin levels (INS), insulin resistance (HOMA‐IR) and HOMA‐B measures), lipid profile (serum cholesterol, VLDL‐cholesterol concentrations, and total cholesterol/HDL levels), inflammation markers (TNF, and IL‐6). However, there was a significant increase in plasma MDA and TAC levels after taking probiotics compared to the control. Many trials are consistent with our findings,^1^, ^8^, ^21^, ^30^, ^40^, ^41^, ^42^ even though there are also some trials reporting no beneficial effects for probiotics.^24^, ^27^, ^28^ This is probably due to the limited number of studies, small sample sizes, and different types or doses of probiotics. Many studies have shown that consuming probiotics might exert positive effects on metabolic parameters. But the mechanism of action of probiotics is not exactly known. In our meta‐analysis, probiotics could affect significantly on FBG, INS, HOMA‐IR, HOMA‐B, and QUIKI, but have no significant differences on HbA1c, and C‐peptide, with no or high heterogeneity among studies. It may be due to insufficient clinical information of the unknown postprandial blood glucose level, probiotic species, and doses, formulation of probiotic/synbiotic, small sample size, or short duration of the study. Based on the results of other studies, probiotics might improve glycemic and triglyceride homeostasis through effecting signaling line of insulin secretion and lipid profile. The production of SCFAs by probiotics leads to an increase in GLP‐1 secretion, which in turn improves glucose levels through different ways including: (a) stimulating insulin secretion and delaying gastric emptying,^43^ (b) modulating the expression of specific genes essential for glucose metabolism including leptin and grehlin hormonal genes, glucose transporter type 4, glucose‐6‐ phosphatase, and PPAR‐gamma genes,^44^ and finally (c) decreasing toll‐like receptor activity, which in turn enhances insulin sensitivity in muscle.^45^ High heterogeneity observed among studies may be because participants with a range of demographics with various forms of metabolic disease. Also, trial participants represented a range of demographics with various forms of metabolic disease including GDM, hypercholesterolemia, and T2DM,^46^ which was likely to have contributed to the large interstudy heterogeneity observed.

There were no significant differences on lipid profile in LDL‐C, HDL, TG, Chol, and total Chol/HDL but have significant differences on VLDL (*p* = 0.02), with no or low heterogeneity among studies. Probiotics may decrease VLDL cholesterol by suppressing the expression of the nuclear factor (NF)‐kappa light‐chain enhancer of the activated cell pathway. The impact of probiotics on profile lipid depended on a variety of factor such as longer treatment durations, and certain probiotic strains, regular consumption of probiotic, dosage of probiotic, mean age of participants, and lifestyle.^46^ The possible mechanisms in regulating lipid profile homeostasis by probiotics are (a) the action of the bile‐salt hydrolase (BSH) enzyme,^47^ (b) Assimilation of cholesterol into the cell walls of probiotics, (c) production of short‐chain fatty acids,^48^ (d) conversion of cholesterol into coprostanol,^49^ (e) Suppressing the expression of the nuclear factor (NF)‐kappa light‐chain,^50^ (f) Alleviating the expression of the pro‐inflammatory cytokines, and (g) altering the energy pathways of fatty‐acid oxidation.^51^ Some trials and meta‐analysis have reported that probiotic co‐supplementation with other dietary supplements such as omega‐3 and zinc had a superior effect on glycemic and lipid hemostasis.^21^, ^52^ GD or maternal hyperglycemia is associated with increase in the oxidative stress which occurs through increased production of free radicals and pro‐inflammatory cytokines, which not only elevate the risk of patho‐physiological complications such as congenital anomalies, spontaneous abortions, preeclampsia, fetal growth restriction, preterm labor, and low birth weight, also have been linked to various states of insulin resistance.^53^ Pro‐inflammatory cytokines interfere with insulin signaling related to insulin resistance in women with GD resulting in increased inflammatory markers such as C‐reactive protein.^54^ Several studies have shown that probiotics can increase the activity of anti‐oxidative enzymes or modulate the circulatory oxidative stress in women with GD.^7^, ^8^, ^21^, ^41^ This review showed a significant increase in MDA and TAC plasma levels, but did not have a substantial impact on GSH, NO, and CRP levels in women with GD after taking probiotics. Only 1 of the 28 trials included in this review had evaluated the effects of probiotics on pro‐inflammatory cytokines such as IL‐6 and TNF.^24^ Other studies had shown that specific strains of probiotics significantly increase the concentrations of anti‐inflammatory or antioxidant biomarkers such as reactive plasma oxygen metabolites, TAC, MDA, GSH, h‐CRP, T‐AOC, SOD, and TNF‐α.^7^, ^8^, ^21^, ^41^ However, there are inconsistent reports on the beneficial effects of probiotics on serum markers levels.^5^, ^55^, ^56^ Such discrepancies between studies could also have been due be variations in different aspects of probiotic intervention, diagnostic criteria, combination of diets, study design, sample sizes, as well as the differences in the genetic and gut microflora compositions of the study cases. The exact mechanisms through which synbiotics and probiotics exert their anti‐oxidative properties remain largely unknown; but (a) preventing and reducing ascorbate auto‐oxidation and metal ion chelation, (b) reducing the activity of superoxide anion radicals, hydrogen peroxide and reactive oxygen species, (c) preventing the formation of lipid hydro‐peroxides, and (d) improving the anti‐inflammatory factors through production of SCFA in the gut are some of the probable mechanisms.^57^, ^58^, ^59^, ^60^ Studies have reported a relationship between reduction of serum adiponectin concentrations and the risk of GD which might be due to the reduction of insulin sensitivity and anti‐inflammatory effects.^61^, ^62^

Adiponectin is an adipocyte‐derived polypeptide hormone which, following binding to its receptor in the hypothalamus, exerts its anti‐diabetic via regulation of glucose and lipid metabolisms.^63^ It reduces insulin sensitivity by increase of glucose utilization and fatty acid oxidation in skeletal Muscles and liver and to improve glucose tolerance by decreasing hepatic gluconeogenesis, independent of AMPK, decreasing glucose production and improving glycemia control.^64^, ^65^ Adiponectin exerts both anti‐ and pro‐inflammatory effects by expression of proinflammatory cytokines in adipocytes and macrophages.^66^ One study in this meta‐analysis showed that probiotics increase adiponectin concentration in the colostrum,^22^ which may regulate adipokine expression and the inflammatory response.^67^ There are conflicting studies on the effect of prebiotics or synbiotics on adiponectin concentrations.^68^, ^69^ One meta‐analysis study has shown that probiotics have no significant effects on adiponectin and leptin levels in adults.^70^ Four out of the 28 trials reported beneficial effects of probiotics on hypertensive disorders.^5^, ^7^, ^27^, ^71^ Regulation of renin‐angiotensin system via the release of bioactive peptides including angiotensin‐converting enzyme inhibitory peptides,^72^ improvement of lipid profile or blood cholesterol via increased lipolysis and reduction of lipoprotein lipase activity,^73^ improving the blood pressure (BP),^74^ reducing PAI‐1 levels, and decreasing the plasma glucose levels,^14^ are the probable mechanisms probiotics employ to improve hypertensive disorders. Several trials in this review evaluated the effects of probiotics on neonatal outcomes including: macrosomia, birth weight, length, infant hypoglycemia, and hyperbilirubinemia.^7^, ^21^, ^27^, ^30^, ^71^ The effect of probiotics in reducing the severity of neonatal hyperbilirubinemia has been reported.^75^, ^76^ Chen et al.’s^77^ review showed that controlling hyperbilirubinemia by probiotics might occur thorough changes in the intestinal microflora, suppressing the growth of pathogenic bacteria, decreasing the enterohepatic circulation, inhibition of the β‐glucuronidase activity, enhancing the tight junction proteins, and increasing the polyamines level. These results may contradict the findings of other studies.^67^ Inconsistencies in the findings of different studies might be due to variations in the study design, sample size, geographical locations, participant selection criteria, participants’ age, and blood pressure levels at the time of sample collection.

Probiotics can improve metabolic syndrome because of their influential impact on the gut microbiota. However, the detailed mechanism for this relation is not clearly understood. There are numerous studies which have analyzed the gut microbiota composition in women with GD, but data in this regard are yet inconsistent. A shift in the composition of the gut microbial during pregnancy is induced by pregnancy hormones, types of nutrition, maternal obesity, delivery mode, and ethnicity.^78^ These changes cause inflammation and are related with obesity or adiposity, blood glucose, hypertensive disorders (including pre‐eclampsia, pregnancy‐induced hypertension, eclampsia), insulin resistance, and circulating pro‐inflammatory cytokines in the pregnant mother, which in overall, affect the mother and infant health.^79^, ^80^ The abundance of certain microorganisms in the gastrointestinal tract such as those in the Firmicutes, Proteobacteria, Bacteroidetes, and Actinobacteria phyla, including Ruminococcaceae, *Desulfovibrio*, Enterobacteriaceae, *P*. *distasonis*, *Prevotella*, and *Collinsella* is involved in the progression of metabolic disorders or GD during pregnancy.^81^ The normal flora of the intestine can modify almost 10% of the host's transcriptome, particularly genes related to immune system response, cellular proliferation, and metabolism.^82^ SCFAs and butyrate are generated as end products of fermentation of dietary fibers by probiotics. Binding to their receptors on enteroendocrine cells in the gut, these products can alter the metabolic pathways responsible for metabolic syndrome and satiety.^83^ On other hand, probiotics improve epithelial barrier function by increasing the levels of adhesion proteins, such as E‐cadherin and β‐catenin, Trials included in this review used different probiotic strains which have different effects on the composition, diversity, and function of the microbiota, and hence, have various effects on the metabolic function of the host.^14^, ^15^ No evaluation of the safety of probiotics was performed in these trials. Chen et al. showed that probiotics are not only effective for the treatment of neonatal jaundice but have no side effects.^84^

Overall, there are several constraints that should be acknowledged in this meta‐analysis: (a) the bias in the included studies due to the small number of available trials and small sample size which could affect the final results; (b) variation in the methods and probiotics preparation protocols, types of species, number of probiotic strains, and the dosage of probiotics used and mean age of participants among trials which can be the reason for different effects of probiotics on the glucose and glycemic factors; (c) length of the interventions used in some studies were not obvious [some studies used short duration of intervention (e.g., 4–8 weeks) which might be insufficient to induce effects in women with GD]; (d) the follow‐up duration was short in some trials; (e) different stages of gestation among participants which can be a confounding factor for pooling the studies. These factors may have increased heterogeneity among the studies; (f) very few studies included in this meta‐analysis assessed the effect of probiotics on the characteristics of mothers and infant, such as pre‐pregnancy and pregnancy body weight, BMI, smoking habits, delivery type, the gestational age at birth, macrosomia, and the presence of neonatal hyperglycemia; (g) other limitation like exclusion of unpublished study data in this research might have led to bias to the pooled effect. Our meta‐analysis had some strength as well. A large number of studies have been reviewed since 2010, with most of the trials at a low risk of bias. The present meta‐analysis also investigated a large number of biomarkers, factors, and outcomes compared to other previous meta‐ analysis, including the type of intervention (probiotics or synbiotics), dosages, as well as the length of intervention, and follow‐up periods among the pregnant women. Since most of the studies were from different geographical areas, unlike other studies, the results of this study can be generalized to some extent. Further research needs to clarity optimal species, dose and duration of intervention in these patients. Also, more randomized trials with larger sample size, different races of participants, and focus on important outcomes in pregnant women and neonates are needed to validate the beneficial effects and safety of probiotics in women with GD.

## 5 CONCLUSION

Despite the presence of heterogeneity and intervening factors among the existing studies, we could discreetly declare that probiotic supplementation, through regulation of the gut microbiota, seems to be able to improve the immune system function, glucose and lipid metabolisms, inflammation, and oxidative stress and subsequently reduce the risk of GD among pregnant women. But above findings remain uncertain, due to the heterogeneity among existing studies. However, more homogeneous studies are needed to confidently generalize the results of this study. Therefore, specific probiotics supplementations may be introduced as one of the adjuvant therapies for GD patients.

## CONFLICT OF INTEREST

Authors declare that there is no conflict of interest.

## AUTHOR CONTRIBUTIONS

AD and RG conceived, designed, and supervised the study. MM and RA contributed to data collection, interpretation, and final approval of data for the work. TF and ST developed the first and final draft of the manuscript. PA and MM developed the second draft of the manuscript. All figures and tables were designed and checked by FV and PA. All authors reviewed and contributed to the revisions and finalized the drafts.

## Supporting information

File S1

File S2

File S3

## Data Availability

All relevant data are included in the manuscript.
