# Supplementary material for: The effect of probiotics on gestational diabetes and its complications in pregnant mother and newborn: A systematic review and meta‐analysis during 2010–2020
Source: J Clin Lab Anal. 2022 Mar 3;36(4):e24326. doi: 10.1002/jcla.24326 (PMC8993604; doi:10.1002/jcla.24326)
Supplement: Supplementary file 2 — File S2 [file JCLA-36-e24326-s003.docx]

**Supplementary File 1**

**Supplementary file 1:** Detailed procedures for the systematic review including its search queries.

1. ***Research methodology***

This systematic review was developed based on the PRISMA guidelines (Preferred Reporting Items for Systematic reviews and Meta-analysis), composed of a checklist of 27 items and a four-step flowchart to guide the review.

**
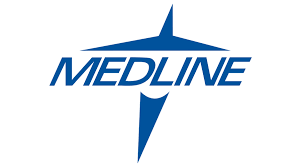
**Searches were made 2010 to 2020 in the scientific databases.

1. **
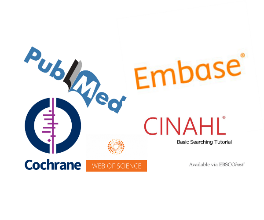
 Medline**, database of the National Library of Medicine, using the following descriptors Medical Subject Headings (MeSH): “*gestational diabetes*”,  “*probiotics*”.
2. **Embase**, database EMBASE is the European counterpart of MEDLINE and contains over 32 million references
3. **
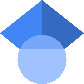

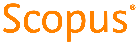
Scopus** is Elsevier’s abstract and citation database launched in 2004. Scopus covers nearly 36,377 titles from approximately 11,678 publishers, of which 34,346 are peer-reviewed journals in top-level subject fields: life sciences, social sciences, physical sciences and health sciences.
4. **Google scholor** provides a simple way to broadly search for scholarly literature.
5. **Detailed searches in**

- **Medline**
- ***Free Search***

" *gestational diabetes*" AND "*probiotics*"

Filters: **Randomized Controlled Trial, Humans**

- ***Indexed Search***
- " *gestational diabetes*"[Mesh] AND "*probiotics*"[Mesh]

Filters: **Randomized Controlled Trial, Humans**

- **Embase**
- ***Free* search**

('gestational diabetes' OR 'impaired glucose tolerance') AND ('probiotic agent') AND [randomized controlled trial]

- ***Indexed Search***

'probiotic agent'/exp/mj AND (' Mouthwashes'/exp/mj OR 'impaired glucose tolerance/exp/mj) AND [randomized controlled trial]/lim AND English:la

- **Google** **scholor**

*“gestational diabetes”* *probiotics*

Return articles dated between 2010-2020

- **Scopus**

TITLE-ABS-KEY ( "*gestational diabetes*" )  AND  TITLE-ABS KEY ( "probiotics" )  AND  ( LIMIT-TO ( DOCTYPE ,  "ar"  ) )  AND  ( LIMIT-TO ( LANGUAGE ,  "English" )
