# Supplementary material for: The effect of probiotics on gestational diabetes and its complications in pregnant mother and newborn: A systematic review and meta‐analysis during 2010–2020
Source: J Clin Lab Anal. 2022 Mar 3;36(4):e24326. doi: 10.1002/jcla.24326 (PMC8993604; doi:10.1002/jcla.24326)
Supplement: Supplementary file 3 — File S3 [file JCLA-36-e24326-s001.docx]

**Supplementary file 3**

Table S1: quality assessment using a Joanna Briggs Institute (JBI) Critical Appraisal Checklist tool for studies included in the systematic review.

| Questions/  Author | 1. Was true randomization used for assignment of participants to treatment groups? | 2. Was allocation to treatment groups concealed? | 3. Were treatment groups similar at the baseline? | 4. Were participants blind to treatment assignment? | 5. Were those delivering treatment blind to treatment assignment? | 6. Were outcomes assessors blind to treatment assignment? | 7. Were treatment groups treated identically other than the intervention of interest? | 8. Was follow up complete and if not, were differences between groups in terms of their follow up adequately described and analyzed? | 9. Were participants analyzed in the groups to which they were randomized? | 10. Were outcomes measured in the same way for treatment groups? | 11. Were outcomes measured in a reliable way? | 12. Was appropriate statistical analysis used? | 13. Was the trial design appropriate, and any deviations from the standard RCT design (individual randomization, parallel groups) accounted for in the conduct and analysis of the trial? | Quality assessment |
| --- | --- | --- | --- | --- | --- | --- | --- | --- | --- | --- | --- | --- | --- | --- |
| Allen,  2010 (1) | Yes | Yes | Yes | Yes | Yes | No | Yes | Yes | Yes | Yes | Yes | Yes | Yes | High |
| Jafarnejad,  2016 (2) | Yes | Yes | Yes | Yes | Yes | No | Yes | Yes | Yes | Yes | Yes | Yes | Yes | High |
| Mehri Jamilian,  2018 (3) | Yes | Yes | Yes | Yes | No | No | Yes | Yes | Yes | Yes | Yes | Yes | Yes | High |
| Shahnaz Ahmadi,  2016 (4) | Yes | Yes | Yes | Yes | No | No | Yes | Yes | Yes | Yes | Yes | Yes | Yes | High |
| Z Asemi,  2012 (5) | Yes | Yes | Yes | No | No | No | Yes | Yes | Yes | Yes | Yes | Yes | No | High |
| Bita Badehnoosh,2017 (6) | Yes | Yes | Yes | No | No | No | Yes | Yes | Yes | Yes | Yes | Yes | No | High |
| Leonie K. Callaway,  2019 (7) | Yes | Yes | Yes | No | No | No | Yes | Yes | Yes | Yes | Yes | Yes | No | High |
| Neda Dolatkhah,  2015 (8) | Yes | No | Yes | Yes | No | No | Yes | Unclear | Yes | Yes | Yes | Yes | No | High |
| Majid Hajifaraji,  2017 (9) | Yes | Yes | Yes | Yes | No | No | Yes | Unclear | Yes | Yes | Yes | Yes | Yes | High |
| Maryam Karamali,  2017 (10) | Yes | Yes | Yes | No | No | No | Yes | Yes | Yes | Yes | Yes | Yes | No | High |
| Athasit Kijmanawat,2019 (11) | Yes | No | Yes | No | No | No | Yes | Yes | Yes | Yes | Yes | Yes | No | High |
| Karen L. Lindsay,  2015 (12) | Yes | Yes | Yes | Yes | No | Yes | Yes | Yes | Yes | Yes | Yes | Yes | Yes | High |
| Zohoor Nabhani,  2018 (13) | Yes | No | Yes | Yes | Yes | Yes | Yes | Yes | Yes | Yes | Yes | Yes | Yes | High |
| Marloes Dekker Nitert1,  2013 (14) | Yes | Yes | Yes | Yes | Yes | Yes | Yes | Yes | Yes | Yes | Yes | Yes | Yes | High |
| Outi Pellonper¨a,  2019 (15) | Yes | Yes | Yes | Yes | Yes | Yes | Yes | Yes | Yes | Yes | Yes | Yes | Yes | High |
| Kristin L. Wickens,  2017 (16) | Yes | Yes | Yes | Yes | No | Yes | Yes | Yes | Yes | Yes | Yes | Yes | Yes | High |
| Hanieh Asgharian,  2019 (17) | Yes | Yes | Yes | Yes | Yes | Yes | Yes | Yes | Yes | Yes | Yes | Yes | Yes | High |
| Mahtab Babadi,  2018 (18) | Yes | Yes | Yes | Yes | No | Yes | Yes | Yes | Yes | Yes | Yes | Yes | Yes | High |
| Christine Barthow,  2016 (19) | Yes | Yes | Yes | Yes | Yes | Yes | Yes | Yes | Yes | Yes | Yes | Yes | Yes | High |
| Luisa F. Gomez-Arango,  2016 (20) | Yes | No | Yes | No | No | No | Yes | Yes | Yes | Yes | Yes | Yes | No | High |
| Luisa F. Gomez-Arango,  2017 (21) | Yes | No | Yes | No | No | No | Yes | Yes | Yes | Yes | Yes | Yes | No | High |
| Sofie Ingdam Halkjaer,  2016 (22) | Yes | Yes | Yes | Yes | Yes | Yes | Yes | Yes | Yes | Yes | Yes | Yes | Yes | High |
| Karen L Lindsay,  2014 (23) | Yes | Yes | Yes | Yes | Yes | Yes | Yes | Yes | Yes | Yes | Yes | Yes | Yes | High |
| Raakel Luoto,  2012 (24) | Yes | Yes | Yes | Yes | Yes | Yes | Yes | Yes | Yes | Yes | Yes | Yes | Yes | High |
| Farnaz Sahhaf Ebrahimi,  2019 (25) | Yes | Yes | Yes | Yes | Yes | Yes | Yes | Yes | Yes | Yes | Yes | Yes | Yes | High |
| Lihui Si,  2019 (26) | Yes | Yes | Yes | Yes | Yes | Yes | Yes | Yes | Yes | Yes | Yes | Yes | Yes | High |
| Shaun Sabico,  2017 (27) | Yes | Yes | Yes | Yes | Yes | No | Yes | Yes | Yes | Yes | Yes | Yes | Yes | High |
| Maryam Karamali,  2018 (28) | Yes | Yes | Yes | Yes | Yes | Yes | Yes | Yes | Yes | Yes | Yes | Yes | Yes | High |

**Reference:**

1. Allen SJ, Jordan S, Storey M, Thornton CA, Gravenor M, Garaiova I, et al. Dietary supplementation with lactobacilli and bifidobacteria is well tolerated and not associated with adverse events during late pregnancy and early infancy. The Journal of nutrition. 2010;140(3):483-8.

2. Jafarnejad S, Saremi S, Jafarnejad F, Arab A. Effects of a Multispecies Probiotic Mixture on Glycemic Control and Inflammatory Status in Women with Gestational Diabetes: A Randomized Controlled Clinical Trial. Journal of Nutrition and Metabolism. 2016;2016:1-8.

3. Jamilian M, Amirani E, Asemi Z. The effects of vitamin D and probiotic co-supplementation on glucose homeostasis, inflammation, oxidative stress and pregnancy outcomes in gestational diabetes: A randomized, double-blind, placebo-controlled trial. Clinical nutrition (Edinburgh, Scotland). 2019;38(5):2098-105.

4. Ahmadi S, Jamilian M, Tajabadi-Ebrahimi M, Jafari P, Asemi Z. The effects of synbiotic supplementation on markers of insulin metabolism and lipid profiles in gestational diabetes: a randomised, double-blind, placebo-controlled trial. The British journal of nutrition. 2016;116(8):1394-401.

5. Asemi Z, Samimi M, Tabassi Z, Naghibi Rad M, Rahimi Foroushani A, Khorammian H, et al. Effect of daily consumption of probiotic yoghurt on insulin resistance in pregnant women: a randomized controlled trial. European journal of clinical nutrition. 2013;67(1):71-4.

6. Badehnoosh B, Karamali M, Zarrati M, Jamilian M, Bahmani F, Tajabadi-Ebrahimi M, et al. The effects of probiotic supplementation on biomarkers of inflammation, oxidative stress and pregnancy outcomes in gestational diabetes. The Journal of Maternal-Fetal & Neonatal Medicine. 2018;31(9):1128-36.

7. Callaway L, McIntyre H, Barrett H, Foxcroft K, Tremellen A, Lingwood B, et al. Probiotics for the Prevention of Gestational Diabetes Mellitus in Overweight and Obese Women: Findings From the SPRING Double-Blind Randomized Controlled Trial. Diabetes Care. 2019;42:dc182248.

8. Dolatkhah N, Hajifaraji M, Abbasalizadeh F, Aghamohammadzadeh N, Mehrabi Y, Abbasi MM. Is there a value for probiotic supplements in gestational diabetes mellitus? A randomized clinical trial. Journal of health, population, and nutrition. 2015;33:25.

9. Hajifaraji M, Jahanjou F, Abbasalizadeh F, Aghamohammadzadeh N, Mesgari Abbasi M, Dolatkhah N. Effect of probiotic supplements in women with gestational diabetes mellitus on inflammation and oxidative stress biomarkers: A randomized clinical trial. Asia Pacific journal of clinical nutrition. 2018;27:581-91.

10. Karamali M, Dadkhah F, Sadrkhanlou M, Jamilian M, Ahmadi S, Tajabadi-Ebrahimi M, et al. Effects of probiotic supplementation on glycaemic control and lipid profiles in gestational diabetes: A randomized, double-blind, placebo-controlled trial. Diabetes & metabolism. 2016;42(4):234-41.

11. Kijmanawat A, Panburana P, Reutrakul S, Tangshewinsirikul C. The effects of probiotic supplements on insulin resistance in gestational diabetes mellitus: a double-blind randomized controlled trial. Journal of Diabetes Investigation. 2018;10.

12. Lindsay K, Brennan L, Kennelly M, Maguire O, Smith T, Curran S, et al. Impact of probiotics in women with gestational diabetes mellitus on metabolic health: A randomized controlled trial. American journal of obstetrics and gynecology. 2015;212.

13. Nabhani Z, Hezaveh SJG, Razmpoosh E, Asghari-Jafarabadi M, Gargari BP. The effects of synbiotic supplementation on insulin resistance/sensitivity, lipid profile and total antioxidant capacity in women with gestational diabetes mellitus: A randomized double blind placebo controlled clinical trial. Diabetes research and clinical practice. 2018;138:149-57.

14. Nitert MD, Barrett HL, Foxcroft K, Tremellen A, Wilkinson S, Lingwood B, et al. SPRING: an RCT study of probiotics in the prevention of gestational diabetes mellitus in overweight and obese women. BMC Pregnancy Childbirth. 2013;13:50.

15. Pellonperä O, Mokkala K, Houttu N, Vahlberg T, Koivuniemi E, Tertti K, et al. Efficacy of Fish Oil and/or Probiotic Intervention on the Incidence of Gestational Diabetes Mellitus in an At-Risk Group of Overweight and Obese Women: A Randomized, Placebo-Controlled, Double-Blind Clinical Trial. Diabetes care. 2019;42(6):1009-17.

16. Wickens K, Barthow C, Murphy R, Abels P, Maude R, Stone P, et al. Early pregnancy probiotic supplementation with Lactobacillus rhamnosus HN001 may reduce the prevalence of gestational diabetes mellitus: a randomised controlled trial. The British journal of nutrition. 2017;117:1-10.

17. Asgharian H, Homayouni-Rad A, Mirghafourvand M, Mohammad-Alizadeh-Charandabi S. Effect of probiotic yoghurt on plasma glucose in overweight and obese pregnant women: a randomized controlled clinical trial. European journal of nutrition. 2020;59(1):205-15.

18. Babadi M, Khorshidi A, Aghadavood E, Samimi M, Kavossian E, Bahmani F, et al. The Effects of Probiotic Supplementation on Genetic and Metabolic Profiles in Patients with Gestational Diabetes Mellitus: a Randomized, Double-Blind, Placebo-Controlled Trial. Probiotics and antimicrobial proteins. 2019;11(4):1227-35.

19. Barthow C, Wickens K, Stanley T, Mitchell E, Maude R, Abels P, et al. The Probiotics in Pregnancy Study (PiP Study): Rationale and design of a double-blind randomised controlled trial to improve maternal health during pregnancy and prevent infant eczema and allergy. BMC Pregnancy and Childbirth. 2016;16:133.

20. Gomez Arango L, Barrett H, McIntyre H, Callaway L, Morrison M, Nitert M. Increased Systolic and Diastolic Blood Pressure Is Associated With Altered Gut Microbiota Composition and Butyrate Production in Early Pregnancy. Hypertension. 2016;68:HYPERTENSIONAHA.116.07910.

21. Gomez Arango L, Barrett H, Wilkinson S, Callaway L, McIntyre H, Morrison M, et al. Low dietary fiber intake increases Collinsella abundance in the gut microbiota of overweight and obese pregnant women. Gut Microbes. 2017;9:00-.

22. Halkjaer SI, Nilas L, Carlsen EM, Cortes D, Halldórsson TI, Olsen SF, et al. Effects of probiotics (Vivomixx®) in obese pregnant women and their newborn: study protocol for a randomized controlled trial. Trials. 2016;17(1):491.

23. Lindsay K, Kennelly M, Culliton M, Smith T, Maguire O, Shanahan F, et al. Probiotics in obese pregnancy do not reduce maternal fasting glucose: A double-blind, placebo-controlled, randomized trial (Probiotics in Pregnancy Study). The American journal of clinical nutrition. 2014;99.

24. Luoto R, Laitinen K, Nermes M, Isolauri E. Impact of maternal probiotic-supplemented dietary counseling during pregnancy on colostrum adiponectin concentration: A prospective, randomized, placebo-controlled study. Early human development. 2011;88:339-44.

25. Sahhaf F, Homayouni A, Mosen M, Abbasalizadeh F, Tabrizi A, Khalili L. Effect of L. acidophilus and B. lactis on blood glucose in women with gestational diabetes mellitus: A randomized placebo-controlled trial. Diabetology & Metabolic Syndrome. 2019;11.

26. Si L, Lin R, Jia Y, Jian W, Yu Q, Wang M, et al. Lactobacillus bulgaricus improves antioxidant capacity of black garlic in the prevention of gestational diabetes mellitus: a randomized control trial. Bioscience reports. 2019;39(8).

27. Sabico S, Al-Mashharawi A, Al-Daghri NM, Wani K, Amer OE, Hussain DS, et al. Effects of a 6-month multi-strain probiotics supplementation in endotoxemic, inflammatory and cardiometabolic status of T2DM patients: A randomized, double-blind, placebo-controlled trial. Clinical nutrition (Edinburgh, Scotland). 2019;38(4):1561-9.

28. Karamali M, Nasiri N, Taghavi Shavazi N, Jamilian M, Bahmani F, Tajabadi-Ebrahimi M, et al. The Effects of Synbiotic Supplementation on Pregnancy Outcomes in Gestational Diabetes. Probiotics and antimicrobial proteins. 2018;10(3):496-503.
